# Emotional wellbeing in neurodivergent populations

**DOI:** 10.3389/fpsyg.2025.1606232

**Published:** 2025-06-30

**Authors:** J. David Pincus, Ken Beller

**Affiliations:** ^1^Leading Indicator Systems, Boston, MA, United States; ^2^Near Bridge, Inc., Sedona, AZ, United States

**Keywords:** neurodivergence, neurodiversity, unified model (UM), emotional needs (EN), image-based assessment, motivation, emotions, neurodivergent

## Abstract

**Introduction:**

Understanding emotional wellbeing in neurodivergent populations remains a critical yet underexplored area in psychological research.

**Methods:**

This study employs AgileBrain, a novel assessment rooted in a neuroscience-informed model of human motivation, to evaluate emotional activation, valence, and unmet emotional needs across a large and diverse sample of adults reporting diagnosed neurodivergent conditions. The sample includes individuals self-identifying with ADHD, ASD, DCD, SID, SH, OCD, and other conditions, alongside a neurotypical comparison group.

**Results:**

Findings reveal systematic variations in emotional wellbeing indexed by three key indicators: (1) overall valence (positive vs. negative emotional needs), (2) activation level (intensity of emotional needs), and (3) the resulting wellbeing index (a composite of the first two). Neurotypical respondents exhibited the highest wellbeing, characterized by low activation and positive valence. DCD and ASD groups showed moderate wellbeing with elevated activation, while groups identifying with SID, SH, and OCD exhibited increasingly negative need valence and a steep drop in overall wellbeing. Notably, the largest group—those reporting ADHD—showed moderate activation with a negative need profile, resulting in low overall wellbeing. A final group categorized as “other conditions” (e.g., depression, anxiety, bipolar disorder, PTSD) exhibited the most extreme negativity in need valence and the lowest wellbeing scores.

**Discussion:**

The study demonstrates the value of a needs-based framework for understanding emotional profiles in neurodivergent populations. By going beyond diagnostic labels to quantify emotional need dynamics, this approach offers scalable, quantitative insights into the lived experience of neurodivergent individuals and highlights distinct pathways to improving wellbeing. The results support the potential for targeted interventions grounded in emotional need fulfillment to enhance resilience and support across diverse neurodivergent profiles.

## Introduction

The increasing recognition of neurodiversity in the general population has brought renewed attention to the varied neurological conditions that shape human experience, particularly Autism Spectrum Disorder (ASD), Attention Deficit Hyperactivity Disorder (ADHD), Obsessive-Compulsive Disorder (OCD), and other mental health conditions such as Depression, Anxiety, Post-Traumatic Stress Disorder (PTSD), and Bipolar Disorder.

These conditions, though distinct in their manifestations, share a common impact on the emotional wellbeing of affected individuals (Wilson et al., 2024). As we continue to deepen our understanding of neurodiversity, it becomes increasingly evident that traditional mental health assessment tools, which are often grounded in verbal and cognitive frameworks, fall short in capturing the nuanced emotional experiences of individuals within each of these populations (Newson et al., 2020).

Epidemiology data based on DSM-5 criteria underscores the widespread nature of these conditions. ASD is estimated to affect approximately 2.21% of U.S. adults aged 18 and older, with state prevalence ranging from 1.97 to 2.42% (Dietz et al., 2020). ADHD, affecting around 11.4% of U.S. children, continues into adulthood, with a prevalence of 12.2% in young adults (Danielson et al., 2024). OCD affects approximately 1.3% of adults, with a lifetime prevalence of 2.3% in the general population (Fawcett et al., 2020). Additionally, PTSD affects 8.3% of U.S. adults across their lifetime (Kilpatrick et al., 2013), while anxiety disorders such as Generalized Anxiety Disorder (GAD) have a lifetime prevalence of 3.7% (Ruscio et al., 2017), and Bipolar I Disorder is found in 1.5% of the adult population (Blanco et al., 2017). These conditions, often co-occurring, highlight the need for more effective mental health assessments and interventions tailored to the unique emotional experiences of neurodivergent individuals.

Individuals with ASD, ADHD, OCD, PTSD, GAD, depression disorders, and bipolar disorder consistently face significant challenges in emotional regulation, recognition, and articulation (Newson et al., 2020). These difficulties are often compounded by co-occurring conditions such as alexithymia, which impairs the ability to identify and describe one’s own emotions (Poquérusse et al., 2018). For example, individuals with ASD frequently exhibit higher rates of alexithymia, which, coupled with difficulties in social cognition and the ability to recognize emotions in others, contributes to significant emotional and social challenges (Griffin et al., 2016). Similarly, those with ADHD often experience emotion dysregulation, particularly when recognizing emotions like anger and fear, and rely on maladaptive strategies for managing their emotions (Soler-Gutiérrez et al., 2023). OCD, while primarily characterized by obsessive thoughts and compulsive behaviors, also impairs the ability to access and process emotions, leading to psychological inflexibility and diminished emotional intelligence (Lazarov et al., 2021). PTSD and anxiety disorders are marked by heightened emotional arousal (stress), which impairs the ability to regulate emotions, often exacerbating feelings of anxiety and fear (Kilpatrick et al., 2013; Ruscio et al., 2017). Bipolar disorder presents additional challenges with extreme fluctuations in mood, leading to periods of emotional instability, including manic and depressive episodes, which further complicate emotional processing (Blanco et al., 2017). These emotional challenges underscore the need for more accurate and effective tools to assess and address the authentic emotional and wellbeing states of neurodivergent individuals (Newson et al., 2020).

Traditional mental health assessments, reliant on detailed verbal instructions and cognitively taxing tasks, can exacerbate these challenges. For example, individuals with ASD often struggle with expressive and receptive language, making it difficult to articulate their emotions fully (Tager-Flusberg and Kasari, 2013), and those with ADHD may find it hard to organize thoughts, regulate impulsivity, or maintain focus during lengthy interviews or questionnaires (Barkley, 2014). Moreover, the inconsistency and heterogeneity among existing instruments—ranging from the symptoms they probe to the phrasing of questions—can obscure true emotional functioning, leading to incomplete or distorted wellbeing profiles that complicate both diagnosis and treatment (Newson et al., 2020).

A promising alternative is AgileBrain, an innovative, image-based emotional assessment tool that overcomes many of the limitations of traditional methods. By utilizing a rapid-exposure image selection process, AgileBrain captures emotional responses before cognitive filtering can occur, ensuring a more accurate reflection of emotional states (Pincus, 2024d). Instructions to participants are available through narrated animated videos; the use of written instructions is kept to a minimum, involving a simple prompt sentence (“I wish I could feel a little more.”). AgileBrain’s image-based design, which minimizes both verbal requirements and cognitive load, provides a standardized, engaging framework that captures authentic emotional responses. AgileBrain’s gamified design, with its brief administration time (approximately 3 min), also enhances user engagement and encourages repeated use, making it a valuable tool for monitoring emotional wellbeing over time. The tool has demonstrated construct validity and strong correlations with established clinical measures of depression, anxiety, and loneliness (Pincus, 2024d), positioning it as a robust alternative for assessing emotional wellbeing in neurodivergent individuals.

Based on current research, neurodiverse groups exhibit distinct emotional-need profiles. For example, individuals with Autism Spectrum Disorder often report heightened needs for social inclusion and authenticity, reflecting their challenges in social cognition and reciprocity (Orsmond et al., 2013). In contrast, those with Attention-Deficit/Hyperactivity Disorder typically experience elevated emotional activation around focus and accomplishment, stemming from difficulties in attention regulation and goal attainment (Barkley, 2014; Beheshti et al., 2020). Adults with anxiety disorders and Post-Traumatic Stress Disorder characteristically show increased needs for psychological safety, driven by hypervigilance to threat and heightened fear responses (Etkin and Wager, 2007). Similarly, individuals with Obsessive-Compulsive Disorder often demonstrate pronounced needs for control and certainty, as compulsive behaviors serve to alleviate distress from uncertainty (Stewart et al., 2017).

By employing an image-based assessment like AgileBrain—which minimizes reliance on verbal analytic processing—we anticipate uncovering emotional patterns that traditional self-report tools may overlook, particularly among those who face linguistic or executive-functioning barriers to articulation (Tager-Flusberg and Kasari, 2013). If these hypothesized patterns are confirmed, they could guide the development of tailored interventions that address the specific emotional needs of each neurodivergent group—and even for each individual person.

### Emotional wellbeing defined

Emotional wellbeing can be viewed as a product of need fulfillment. When our emotional needs are fulfilled, we feel emotionally well; when our needs go unfulfilled, we feel emotionally unwell. A recently introduced unified model of human motivation provides a comprehensive framework for understanding these needs, integrating fragmented theories into a single structure based on first principles (Pincus, 2022a) This model organizes motivation across four life domains—Self, Material, Social, and Spiritual—each operating at three levels of striving: Foundational (the most basic needs in each domain), Experiential (needs of becoming built on the fulfillment of foundational needs), and Aspirational (the highest-level needs, built on the satisfaction of foundational and experiential needs, representing the highest outcomes in each domain). The resulting 12 distinct needs represent the core motivational drivers that underlie human behavior and emotional wellbeing. The framework and its proposed emotional needs have been detailed elsewhere (Pincus, 2022a,b, 2023a,b, 2024a,b,c, 2025a,b,c).

A recent literature review has shown that the concept of subjective wellbeing (SWB) can be understood as a summary state of human need fulfillment, aligned with this model of 12 distinct emotional needs (Pincus, 2023a). This literature review synthesizes nearly 100 distinct wellbeing models identified by Linton et al. (2016), representing 191 components of wellbeing, including the Surgeon General, U.S. Department of Health and Human Services (2022) wellbeing framework. Unlike simplistic measures of happiness or contentment, SWB reflects a dynamic interplay of needs and resources, requiring a broad and nuanced assessment approach.

AgileBrain has recently been validated as an effective measure of emotional wellbeing in the general population through comparisons with a range of established clinical assessments, including the PHQ-9, CESD-10, GAD-7, PSS-10, Brief Cope-28, the UCLA Loneliness Scale, and self-reported trauma. By systematically mapping AgileBrain’s emotional need profiles against these clinical measures, these studies confirm its ability to capture meaningful patterns of wellbeing differences, demonstrating its potential as a comprehensive and theory-driven SWB assessment tool (Pincus, 2024d).

By utilizing images rather than text, AgileBrain reduces linguistic and cultural biases, increasing its applicability across diverse populations and settings. Moreover, its design aligns with the neurological time course for emotional processing (Rudrauf et al., 2008, 2009) ensuring that responses are captured before cognitive filtering or social desirability biases can distort them. This methodological innovation holds particular promise for neurodiverse populations, where conventional assessment tools may fail to accurately reflect emotional experiences due to verbal processing differences or heightened cognitive demands (Newson et al., 2020).

## Method

### Participants

Participants were recruited from a population-representative sample of full-time U.S.-based employees through a professional research panel company. The survey was administered via InnovateMR, LLC, a commercial survey panel that adheres to its own ethical review processes and guidelines, eliminating the need for separate ethics approval. Data collection occurred in June 2023.

### Sample characteristics

The sample was structured to match Bureau of Labor Statistics (BLS) distributions for employment by company size, ensuring representativeness of the U.S. full-time workforce in companies with at least 20 employees. The sample size is n = 1,031 providing margins of error ranging from ±0.61 to ±3.05% at the 95% confidence level. Demographic distributions, including sex, age, and race, closely aligned with the BLS Current Population Survey estimates, ensuring generalizability to the broader U.S. workforce (Table 1).

**Table 1:** Sample characteristics (weighted).

Demographic variable	Bureau of labor statistics^1^	June 2023 survey
Sample size	60,000	1,031
Margin of error at 95% CI	+/− < 1%	+/−3.05%
Response rate	71%	57%
Sex		
Male	56.3%	56.6%
Female	43.7%	43.4%
Age		
18–19	1.0%	0.8%
20–24	7.2%	7.4%
25–34	25.1%	23.9%
35–44	24.4%	24.4%
45–54	23.4%	23.8%
55+	19.0%	19.6%
Race		
Asian (non-Hispanic)	6.6%	6.9%
Black (non-Hispanic)	11.8%	12.9%
Hispanic/Latino	18.5%	19.5%
White (non-Hispanic)	63.0%	60.6%

^1^U.S. Bureau of Labor Statistics (2023). Labor Force Statistics from the Current Population Survey. Demographic characteristics of U.S. workers, employed, usually work full-time, by age, sex, and race.

The unweighted sample size for the neurodivergent sample is n = 336 and n = 661 for the neurotypical sample, giving margins of error of ±5.23% for the neurodivergent sample and ±3.76% for the neurotypical sample at the 95% confidence level (Table 2). Demographic distributions of age and race reflect the increased tendency of younger, White populations to receive neurodivergent diagnoses (Zablotsky et al., 2021); we also observe slight over-representation in the neurodivergent sample among Asian and Hispanic, but not Black, respondents.

**Table 2:** Sample characteristics of neurodivergent and neurotypical respondents (unweighted).

Demographic variable	Neurodivergent sample	Neurotypical sample
Sample size	336	661
Margin of error at 95% CI	±5.23%	+/−3.76%
Male	53.6%	52.9%
Female	46.4%	47.0%
Age (mean)	36.8	42.6
Asian (non-Hispanic)	5.9%	4.1%
Black (non-Hispanic)	17.6%	19.8%
Hispanic/Latino	15.5%	12.7%
White (non-Hispanic)	72.9%	69.3%

### Sample considerations

Given that participants were full-time employees at medium to large organizations, the sample is likely to represent a relatively high-functioning subset of neurodivergent individuals compared to the broader neurodiverse population. This aligns with the research objective of developing an effective, scalable tool for organizations to assess and support the emotional wellbeing of their neurodiverse workforce.

### Procedure

The survey incorporated the AgileBrain image-based assessment to evaluate employees’ emotional need state. The psychometric validity and reliability of AgileBrain, along with details of its protocol, have been previously documented (Pincus, 2023b).

**Figure 1:**
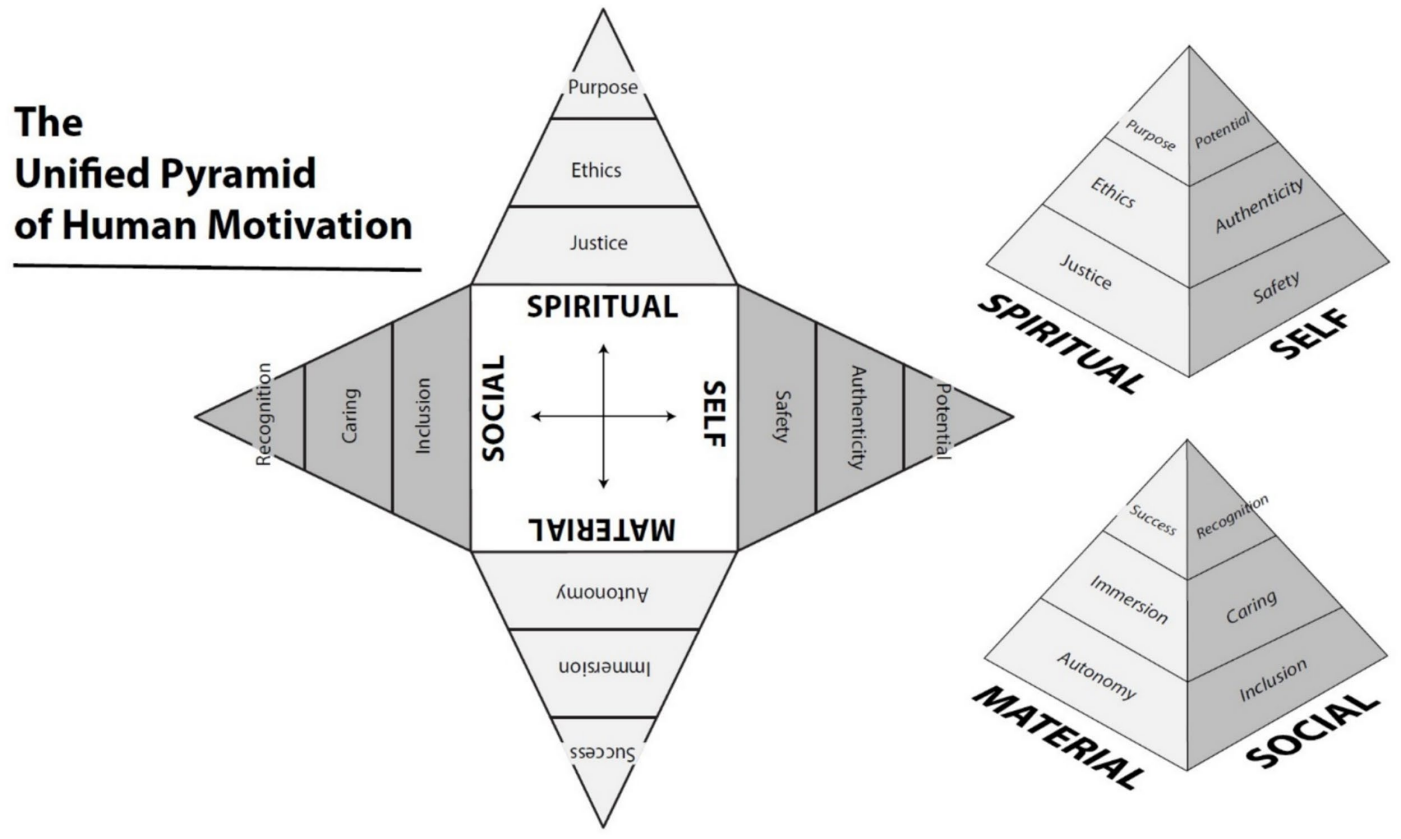
The unified pyramid of human motivation (Pincus, 2022a).

AgileBrain evaluates 12 core emotional needs derived from a unified theory of human motivation (Figure 1). These needs are organized across four life domains—Self, Material, Social, and Spiritual—and three levels of attainment—Foundational (To Be), Experiential (To Do), and Aspirational (To Have). Each need has both a promotion aspect (a state one wishes to feel more of) and a prevention aspect (a state one wishes to feel less of), leading to a total of 24 evaluated emotional states. Promotional forms of each need are listed below without parentheses; corresponding prevention-oriented forms of each need are shown in parentheses below.

- **Self-domain:** Safety (Insecurity), Authenticity (Conformity), Potential (Limitation)
- **Material domain:** Autonomy (Disempowerment), Immersion (Stagnation), Success (Failure)
- **Social domain:** Inclusion (Exclusion), Caring (Uncaring), Recognition (Scorn)
- **Spiritual domain:** Justice (Injustice), Ethics (Wrongdoing), Purpose (Materialism)

Each of these reflects motivational goals tied to unmet emotional needs, with prior theoretical and empirical work validating their distinctiveness.

The images used in AgileBrain are carefully curated photographs validated to represent each emotional need clearly and consistently. Out of an initial set of 320 images sourced from Shutterstock, a rigorous validation process involving 2,728 participants resulted in a final set of 72 images. Each need is represented by three promotion images and three prevention images. Six expert raters classified these images with high inter-rater agreement (Cohen’s *κ* = 0.91). Selection criteria included both visual content analysis and interviews with artists to ensure each image elicited the intended affective association with its target motivation.

Participants complete a rapid image selection task designed to capture affective responses before cognitive interference (such as impression management or rational filtering) occurs. The images are presented for a very short window (500–1,500 milliseconds), during which the participant selects those that match the prompt:

- “Thinking about my current situation, I wish I could feel a little more…” (for promotion needs)
- “Thinking about my current situation, I wish I could feel a little less…” (for prevention needs)

This “gut-level” image selection task bypasses slower rational processing and captures emotionally salient needs based on split-second intuitive judgments.

Image selection indicates the emotional resonance of that image with the participant’s current affective-motivational state. Faster selections are interpreted as stronger emotional responses. Each selected image contributes to a cumulative score for the corresponding emotional need. These scores are aggregated to create three summary-level indices: (1) Activation – the intensity of unmet needs, (2) Valence – the overall emotional positivity or negativity, and (3) Wellbeing Index – a combined measure reflecting emotional health.

Together, these scores form an emotional profile that can be tracked over time or compared across groups.

In this context, “motivations” and “emotional needs” are functionally equivalent. The image validation reported in Pincus (2023b) refers to distinct motivational constructs that map directly onto the 12 emotional needs assessed in AgileBrain. The needs reflect unmet emotional drives that influence behavior, and the images are designed to evoke recognition of those motivational/emotional states. This equivalence is grounded in a model that defines motivation as the emotional striving to fulfill unmet needs.

### Study design and data analysis

This study employed a descriptive, correlational approach using a cross-sectional survey design. Statistical analyses were performed using SPSS 24.0.

### Measures

Data on emotional wellbeing is reported by subgroups representing formally diagnosed neurodiversity conditions.

### Results

#### Diagnosed neurodiversity

Among participants, 34.0% reported having at least one formally diagnosed neurodivergent condition. The prevalence and sample sizes associated with each subgroup are presented in Table 3.

**Table 3:** Estimated incidence and sample sizes for diagnosed neurodiversity conditions (weighted).

Neurodiversity diagnosis^1^	Estimated incidence in full-time working population	Sample size
Attention deficit hyperactivity disorder (ADHD)	19.5%	201
Autism spectrum disorder (ASD)	2.6%	27
Developmental coordination disorder (DCD)	2.6%	27
Obsessive compulsive disorder (OCD)	9.6%	99
Sensory hypersensitivity (SH)	3.5%	36
Sensory integration disorder (SID)	4.2%	43
Other: depression, anxiety, PTSD, bipolar disorder, unspecified	6.9%	71
*Any* neurodiversity diagnosis	34.0%	351
No neurodiversity diagnosis	66.0%	680

^1^Q25. Please select the conditions for which you have been formally diagnosed by a health care professional or mental health professional.

As observed by previous large-scale studies, ASD, DCD, SID are more prevalent in males, whereas OCD, Depression, and Anxiety are more prevalent in females (Satterthwaite et al., 2018). The sex distribution associated with each diagnosed condition are presented in Table 4. Two of the diagnoses (ADHD, OCD) afforded sufficient sample sizes to examine the pattern of need activation by sex. Males with ADHD showed significantly higher need activation than females with ADHD in 10 areas: Insecurity (negative Safety), Conformity (negative Authenticity), Autonomy, Immersion, Success, Caring, Uncaring (negative Caring), Justice, Ethics, Wrongdoing (negative Ethics), and Purpose. Conversely, females with OCD showed significantly higher need activation than males with OCD in 9 areas: Insecurity (negative Safety), Authenticity, Limitation (negative Potential), Autonomy, Immersion, Success, Exclusion (negative Inclusion), Scorn (negative Recognition), and Injustice (negative Justice). This pattern suggests that the emotional challenges experienced by those with ADHD are more pronounced for males than females, whereas emotional difficulties experienced by those of OCD are heightened for females more than males.

**Table 4:** Sex distribution of respondents by neurodivergent diagnosis (unweighted).

Neurodiversity diagnosis^1^	Male	Female
Attention deficit hyperactivity disorder (ADHD)	55.7% (*n* = 107)	44.3% (*n* = 85)
Autism spectrum disorder (ASD)	70.8% (*n* = 17)	29.2% (*n* = 7)
Developmental coordination disorder (DCD)	72.7% (*n* = 16)	27.3% (*n* = 6)
Obsessive compulsive disorder (OCD)	49.4% (*n* = 45)	50.6% (*n* = 46)
Sensory hypersensitivity (SH)	54.8% (*n* = 17)	45.2% (*n* = 14)
Sensory integration disorder (SID)	72.5% (*n* = 29)	27.5% (*n* = 11)
Other: depression, anxiety, PTSD, bipolar disorder, unspecified	39.1% (*n* = 27)	60.9% (*n* = 42)

^1^Q25. Please select the conditions for which you have been formally diagnosed by a health care professional or mental health professional.

#### Emotional wellbeing: aggregate measures

As indicated above, AgileBrain generates three overall scores, in addition to component-level scores, which quantify various aspects of emotional wellbeing (Table 5):

1. Emotional Activation – Represents the intensity of unmet emotional needs as measured by the number of images selected weighted by the latency of selection. More images selected at higher relative speed equates to greater unmet need.
2. Emotional Positivity – Captures the emotional positivity or negativity of a person’s state as measured by the ratio of positive to negative images selected. Relatively greater selection of negative (prevention) images equates to a more negative outlook.
3. Wellbeing^1^ Index – A summary measure calculated as the product of (1 - activation decimal) and positivity. This measure combines activation and positivity/negativity into a single metric.

**Table 5:** AgileBrain activation, positivity, and wellbeing index scores for diagnosed neurodiversity conditions.

Neurodiversity diagnosis^1^	Activation	Positivity	Wellbeing Index
Attention deficit hyperactivity disorder (ADHD)	0.4831	−1.204	−0.62235
Autism spectrum disorder (ASD)	0.5602	−0.1111	−0.04886
Developmental coordination disorder (DCD)	0.5108	0.7778	0.3805
Obsessive compulsive disorder (OCD)	0.4792	−1.0505	−0.5471
Sensory hypersensitivity (SH)	0.5332	−0.8333	−0.38898
Sensory integration disorder (SID)	0.4276	−0.5581	−0.31946
Other: depression, anxiety, PTSD, bipolar disorder, unspecified	0.4883	−4.4789	−2.29185
*Any* neurodiversity diagnosis	0.4093	−0.6456	−0.38136
No neurodiversity diagnosis	0.3888	1.0441	0.638154

^1^Q25. Please select the conditions for which you have been formally diagnosed by a health care professional or mental health professional.

The distribution of activation scores is positively skewed, reflecting the tendency for most individuals in the general population to be more emotionally settled than activated. In contrast, valence scores and the wellbeing index exhibit a normal distribution across the population (for additional details see Pincus, 2024d).

#### Ranking conditions by wellbeing index

**Figure 2:**
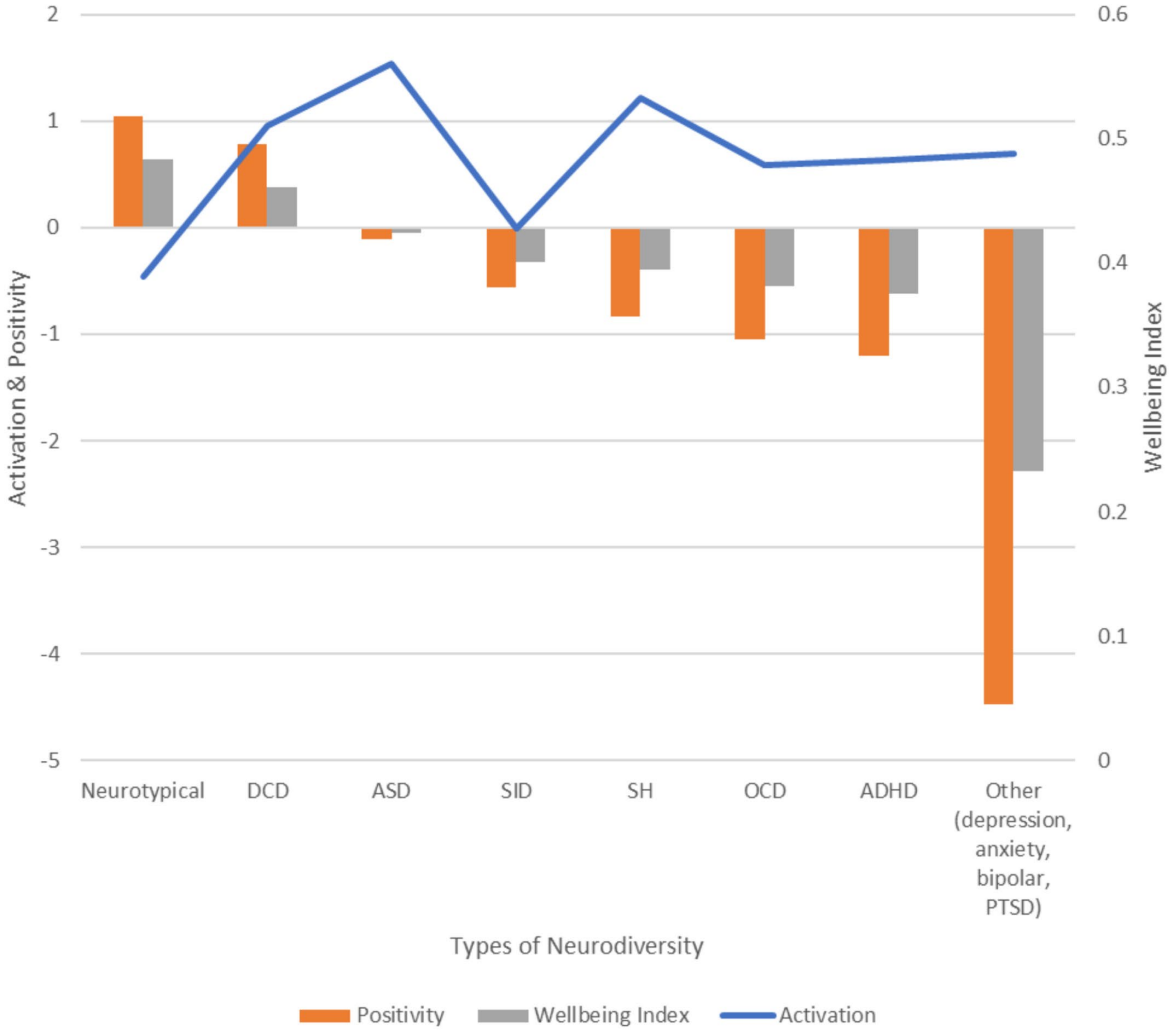
AgileBrain activation, positivity, and wellbeing index scores for diagnosed neurodiversity conditions in descending order of wellbeing index. Neurotypicals show the highest wellbeing, while neurodiverse groups—especially those with mood and trauma-related conditions—exhibit more negative needs and lower wellbeing.

The rank order of diagnosed conditions by the wellbeing index is instructive as a window into the level of emotional distress experienced on average (Figure 2). As expected, respondents with no neurodiversity diagnosis (i.e., neurotypicals) display the highest wellbeing index, which is a function of their relatively low level of activation and relatively high degree of positivity. Working adults with DCD show nearly the same level of wellbeing as neurotypicals, albeit with a higher level of emotional activation. ASD respondents show a wellbeing index that dips slightly negative, which is a function of very high levels of activation or intensity of feeling and slightly negative emotional needs. SID respondents show a much lower level of activation but needs that are more negatively skewed. SH respondents show similarly high levels of activation but a skew toward negative (prevention-oriented) emotional needs. OCD respondents show a reduction in activation combined with increasingly negative needs. ADHD respondents show a level of activation that is similar to that of OCD respondents but needs that are further negative. Respondents citing other conditions, primarily depression, anxiety, bipolar disorder, and PTSD, show slightly elevated activation but the most negative needs by far, and, accordingly, the lowest wellbeing index.

### Emotional wellbeing: specific emotional needs

Independent samples *t*-tests were conducted to compare emotional need fulfillment levels between individuals reporting each neurodiversity diagnosis or symptom and those who did not.

## Results

### Neurotypical

As expected, given that neurotypicals (NTs) represent the baseline psychological cohort, they show the highest wellbeing index. This is due to their combination of low emotional activation and a strong skew toward positive emotional needs when compared to all neurodiverse groups.

NTs showed significantly less activation across the majority of specific needs than respondents who reported at least one diagnosed neurodivergent condition. Of the 24 emotional need measures, 15 showed statistically significantly *lower* activation among NTs, i.e., every unmet need area is more activated among NDs than NTs:

- **Promotion needs:** Authenticity (*p* < 0.001), Autonomy (*p* < 0.001), Immersion (*p* < 0.001), Success (*p* < 0.001), Caring (*p* = 0.023), Ethics (*p* = 0.016), Purpose (*p* = 0.002).
- **Prevention needs:** Insecurity (*p* < 0.001), Conformity (negative Authenticity; *p* = 0.004), Limitation (*p* = 0.012), Exclusion (*p* < 0.001), Uncaring (*p* < 0.001), Scorn (*p* < 0.001), Injustice (negative Justice, *p* = 0.003), Wrongdoing (*p* < 0.001).

By far, the largest mean differences between NTs and NDs occurred in the Social domain (Scorn, Exclusion), followed by Insecurity in the Self domain. In sum, NTs show significantly fewer unmet needs than those reporting at least one neurodiversity condition. The majority of significant differences (53%) involved prevention needs.

### Diagnosed neurodiversities

#### Developmental coordination disorder (DCD, dyspraxia)

Consistent with Harris et al. (2021), AgileBrain data show that individuals with DCD experience reduced emotional positivity alongside heightened emotional activation compared to neurotypical peers.

Out of the 24 emotional need measures, 10 showed statistically significant differences at the *p* < 0.05 level. The majority (70%) are positive, promotional needs for “*more of the good*,” suggesting relatively healthy emotional wellbeing.

Specifically, individuals reporting DCD demonstrated significantly higher activation of the following emotional needs:

- **Promotion needs:** Authenticity (*p* = 0.008), Autonomy (*p* = 0.031), Immersion (*p* = 0.01), Success (*p* = 0.031), Caring (*p* = 0.025), Ethics (*p* = 0.002), Purpose (*p* = 0.002).
- **Prevention needs:** Conformity (negative Authenticity; *p* = 0.004), Scorn (*p* = 0.019), Injustice (negative Justice, *p* = 0.035).

Results by need categories (levels and domains) are summarized in Tables 6–8. In terms of life domains, activation is spread relatively evenly across the four, 20% in the domains of the self and the social, and 30% in the material and spiritual domains. In terms of the three levels of striving, DCD respondents show higher concentration in experiential needs (50%) than the foundational (20%) or aspirational (30%) levels. The largest concentration of *promotion* needs (57%) occurs in the experiential level of striving, and in needs in the material domain (43%).

**Table 6:** Patterns of specific emotional needs distinguishing diagnosed neurodivergent conditions.

	DCD	ASD	SID	SH	OCD	ADHD	Other	Row marginals	Domain means
Safety								0	Self (+) 1.7
Authenticity	1	1		1				3	
Potential			1				1	2	
Autonomy	1	1		1	1	1		5	Material (+) 4.7
Immersion	1	1			1	1		4	
Success	1	1		1	1	1		5	
Inclusion		1		1				2	Social (+) 2.0
Caring	1	1				1		3	
Recognition		1						1	
Justice						1		1	Spiritual (+) 3.0
Ethics	1	1	1	1		1		5	
Purpose	1			1		1		3	
Insecurity				1	1	1	1	4	Self (−) 3.3
Conformity	1	1				1		3	
Limitation		1			1		1	3	
Disempowerment								0	Material (−) 0.3
Stagnation							1	1	
Failure								0	
Exclusion		1		1	1	1		4	Social (−) 4.0
Uncared for					1	1	1	3	
Scorn	1	1		1	1	1		5	
Injustice	1				1		1	3	Spiritual (−) 1.7
Wrongdoing				1		1		2	
Materialism								0	
Column Marginals	10	12	2	10	9	13	6	62	2.6

**Table 7:** Patterns of need category activation distinguishing diagnosed neurodivergent conditions.

		DCD	ASD	SID*	SH	OCD	ADHD	Other	Row means
Level of Striving (share of total needs)	Foundational	20%	25%	0%	40%	44%	31%	33%	28%
	Experiential	50%	42%	50%	30%	22%	46%	33%	39%
	Aspirational	30%	33%	50%	30%	33%	23%	33%	33%
Life domain (share of total needs)	Self	20%	25%	50%	20%	22%	15%	50%	29%
	Material	30%	25%	0%	20%	33%	23%	17%	21%
	Social	20%	42%	0%	30%	33%	31%	17%	25%
	Spiritual	30%	8%	50%	30%	11%	31%	17%	25%
Level of striving (share of promotion needs)	Foundational	14%	25%	0%	33%	33%	29%	0%	19%
	Experiential	57%	50%	50%	33%	33%	43%	0%	38%
	Aspirational	29%	25%	50%	33%	33%	29%	100%	43%
Life domain (share of promotion needs)	Self	14%	13%	50%	17%	0%	0%	100%	28%
	Material	43%	38%	0%	33%	100%	43%	0%	37%
	Social	14%	38%	0%	17%	0%	14%	0%	12%
	Spiritual	29%	13%	50%	33%	0%	43%	0%	24%
Level of Striving (share of prevention needs*)	Foundational	33%	25%	0%	50%	50%	33%	40%	39%
	Experiential	33%	25%	0%	25%	17%	50%	40%	32%
	Aspirational	33%	50%	0%	25%	33%	17%	20%	30%
Life Domain (share of prevention needs*)	Self	33%	50%	0%	25%	33%	33%	40%	36%
	Material	0%	0%	0%	0%	0%	0%	20%	3%
	Social	33%	50%	0%	50%	50%	50%	20%	42%
	Spiritual	33%	0%	0%	25%	17%	17%	20%	19%

*Excluding SID, which was associated with no prevention needs.

**Table 8:** Distinguishing need activations of diagnosed neurodivergent conditions (+ = promotional need, − = prevention need).

		Self	Material	Social	Spiritual
Level of Striving	Aspirational	ASD (−) SID (+) OCD (−) Other (+)	DCD (+) ASD (+) SH (+) OCD (+) ADHD (+)	DCD (−) ASD (+, −) SH (−) OCD (−) ADHD (−)	SH (+) DCD (+) ADHD (+)
	Experiential	DCD (+, −) ASD (+, −) SH (+) ADHD (−)	DCD (+) ASD (+) OCD (+) ADHD (+) Other (−)	DCD (+) ASD (+) OCD (−) ADHD (+, −) Other (−)	DCD (+) ASD (+) SID (+) SH (+, −) ADHD (+, −)
	Foundational	SH (−) OCD (−) ADHD (−) Other (−)	DCD (+) ASD (+) SH (+) OCD (+) ADHD (+)	ASD (+, −) SH (+, −) OCD (−) ADHD (−)	DCD (−) OCD (−) ADHD (+) Other (−)

DCD respondents show a wellbeing index that is nearly as high as neurotypicals, suggesting that this is a group with relatively strong emotional health. The activation of both positive and negative needs related to Authenticity/Conformity suggests that those with movement or balanced disorders internalize their emotions and feel pressure to “fit in” which limits their ability to be accepted as they are (Omer et al., 2018). At least one experiential need is activated in all four domains (Authenticity/Conformity, Immersion, Caring, Ethics), resulting in a high concentration of experiential needs, suggesting that DCD’s negative effects are concentrated on acts of “doing” in-the-moment.

#### Autism spectrum disorder (ASD)

Individuals with ASD typically experience heightened anxiety and emotional challenges in social situations compared to neurotypicals; however, their emotional wellbeing often remains stable or even improves during solitary or non-social activities (Chen et al., 2015).

Out of the 24 emotional need measures, 12 showed statistically significant differences at the *p* < 0.05 level. The majority (67%) are positive, promotional needs for *more of the good,* suggesting relatively healthy emotional wellbeing. ASD respondents show a wellbeing index that dips slightly negative, which is a function of very high levels of activation or intensity of feeling and relatively balanced positive and negative emotional needs.

Specifically, individuals reporting ASD demonstrated significantly higher activation of the following emotional needs:

- **Promotion needs:** Authenticity (*p* = 0.028), Autonomy (*p* = 0.01), Immersion (*p* = 0.01), Success (*p* = 0.01), Inclusion (*p* = 0.031), Caring (*p* = 0.008), Recognition (*p* = 0.021), Ethics (*p* = 0.021).
- **Prevention needs:** Conformity (negative Authenticity; *p* = 0.015), Limitation (negative Potential; *p* = 0.035), Exclusion (negative Inclusion; *p* = 0.049), Scorn (*p* = 0.006).

In terms of life domains, the domain of the self skews negative, with significant differences in needs for less conformity and limitation, and only one positive need (for increased authenticity). All significant material domain needs (autonomy, immersion, success) are associated with promotional needs, suggesting that interactions with the material world are not an area of deficiency for autistic people. Five of the six needs in the social domain are activated, suggesting that this is an area of tension. The only significant difference in the spiritual domain is the need for greater ethics (Dempsey et al., 2020), suggesting that this domain is relatively settled. The largest concentrations of needs occur in the social domain and experiential level of striving (42% each).

#### Sensory integration disorder (SID)

Participants with Sensory Integration Dysfunction (SID) exhibit low emotional activation paired with a slightly negative emotional-needs profile. While standalone research specifically on SID is limited, these findings align closely with known sensory processing challenges commonly seen in ASD, for which SID is a frequent comorbidity. Such sensory difficulties often contribute to increased anxiety, emotional dysregulation, and problems forming secure attachments (Durrani, 2020), explaining why the wellbeing profile for SID closely resembles that of ASD.

Out of the 24 emotional need measures, only two showed statistically significant differences at the *p* < 0.05 level.

- Both (100%) are positive, **promotional** needs for *more of the* good: Potential (*p* < 0.001) and Ethics (*p* = 0.045).

Because this group was not differentiated by any specific unmet prevention needs or foundational needs, and the level of activation relatively low, findings suggest that those with SID have relatively healthy emotional wellbeing.

#### Sensory hypersensitivity (SH)

Participants with sensory hypersensitivity show the second highest levels of activation but a skew toward negative (prevention-oriented) emotional needs and coping styles (Van Reyn et al., 2022).

Out of the 24 emotional need measures, 10 showed statistically significant differences at the *p* < 0.05 level. The majority (60%) are positive, promotional needs for *more of the good,* suggesting relatively healthy emotional wellbeing. SH respondents show a wellbeing index that dips slightly negative, which is a function of very high levels of activation or intensity of feeling and slightly negative emotional needs.

Specifically, individuals reporting SH demonstrated significantly higher activation of the following emotional needs:

- **Promotion needs:** Authenticity (*p* = 0.011), Autonomy (*p* = 0.004), Success (*p* = 0.004), Inclusion (*p* = 0.031), Caring (*p* = 0.008), Ethics (*p* = 0.02), Purpose (*p* < 0.001).
- **Prevention needs:** Insecurity (negative Safety, *p* < 0.001), Exclusion (negative Inclusion; *p* = 0.016), Scorn (*p* = 0.041), Wrongdoing (negative Ethics; *p* = 0.006).

In terms of life domains, activation is spread relatively evenly across the four, 20% in the domains of the self and the material, and 30% in the social and spiritual domains. Needs are also spread relatively evenly across the three levels of striving, with a slightly higher concentration in foundational needs (40%), and 30% in both the experiential and aspirational levels.

Overall, this profile suggests that those with SH experience stress (as indicated by the high level of activation), but that their needs are spread evenly across areas of their lives and are a bit more positive than negative, suggesting that they have reasonably good emotional wellbeing, but highlights the importance of addressing emotional regulation and coping strategies in individuals with sensory hypersensitivity.

#### Obsessive compulsive disorder (OCD)

Consistent with Becker et al. (2014) and Picó-Pérez et al. (2022), OCD participants in this research exhibit slightly elevated activation along with relatively negative emotional needs.

Out of the 24 emotional need measures, 9 showed statistically significant differences at the *p* < 0.05 level. The majority (67%) are negative, prevention-oriented needs for *less of the bad,* suggesting relatively poor emotional wellbeing. OCD respondents show a relatively low wellbeing index, which is a function of moderate levels of activation and a negative emotional need profile.

Specifically, OCD is associated with significantly higher activation of the following emotional needs:

- **Promotion needs:** Autonomy (*p* = 0.037), Immersion (*p* = 0.035), Success (*p* = 0.037).
- **Prevention needs:** Insecurity (negative Safety, *p* = 0.015), Limitation (negative Potential, *p* = 0.046), Exclusion (negative Inclusion; *p* < 0.001), Uncared for (negative Caring; *p* = 0.035), Scorn (negative Recognition, *p* = 0.02), and Injustice (negative Justice, *p* = 0.028).

In terms of life domains, activation is concentrated in the material (33%) and social (33%) domains, with moderate activation in the domain of the self (22%), and mild activation in the spiritual domain (11%). Regarding the three levels of striving, needs are concentrated, respectively, at the foundational (44%), aspirational (33%), and experiential (22%) levels. The largest concentrations of needs occur in *promotion* needs in the material domain, and in *prevention* needs in the social domain and foundational level.

Overall, this profile suggests that those with OCD experience have relatively poor emotional wellbeing with greatest impacts in the social and material (work, play) domains, leading to needs for relief from feelings of Exclusion and Insecurity (both foundational needs).

#### Attention deficit hyperactivity disorder (ADHD)

Attention deficit hyperactivity disorder involves both elevated brain activation to negative emotional stimuli and impaired inhibitory control leading to increased distractibility and emotional dysregulation; these present as heightened sensitivity to negative experiences and functional difficulties (Vetter et al., 2018; Lei et al., 2015). These emotional challenges are evident in both social interactions and personal tasks, contributing to the broader emotional difficulties commonly observed in ADHD (Hirsch et al., 2018).

Out of the 24 emotional need measures, 13 showed statistically significant differences at the *p* < 0.05 level. In terms of valence, needs are relatively evenly split (54% positive; 46% negative), suggesting broad impact.

ADHD respondents exhibit emotional activation that is similar to that of OCD respondents, but with a stronger emphasis on negative emotional needs.

Specifically, individuals reporting attention deficits demonstrated significantly higher activation of the following emotional needs:

- **Promotion needs:** Autonomy (*p* = 0.002), Immersion (*p* < 0.001), Success (*p* = 0.002), Caring (*p* = 0.004), Justice (*p* = 0.022), Ethics (*p* = 0.017), Purpose (*p* = 0.02).
- **Prevention needs:** Insecurity (negative Safety; *p* < 0.001), Conformity (negative Authenticity; *p* = 0.009), Exclusion (negative Inclusion; *p* < 0.001), Uncared for (negative Caring; *p* = 0.006), Scorned (negative Recognition; *p* = 0.001), Wrongdoing (negative Ethics; *p* = 0.002).

The self and social domains skew negative, with significant differences in needs for less Insecurity and Conformity, all three preventative social needs (exclusion, uncared for, scorned).

In sharp contrast, all material and spiritual domain needs are associated with promotional needs, suggesting that interactions with these domains do not represent areas of deficiency for attention challenged people. The largest concentrations of needs occur in *promotion* needs in the material and spiritual domains, and the experiential level of striving, and in *prevention* needs in the social domain and experiential level.

The largest concentration of unmet needs is in the experiential level (46%), indicating that ADHD’s impact is most strongly felt in immediate, task-oriented moments, such as when engaging in social or work-related activities.”

The emotional wellbeing of individuals with ADHD is shaped by heightened emotional activation and significant emotional needs, especially in the Self and Social domains. The unmet needs for autonomy, and for relief from insecurity and social exclusion underscore the emotional struggles that ADHD individuals face, particularly in managing personal relationships and achieving personal goals.

Interventions aimed at emotional regulation, social integration, and goal achievement are crucial to improving the emotional wellbeing of individuals with ADHD. By focusing on these areas, it is possible to help ADHD individuals address their unmet emotional needs and enhance their emotional resilience.

#### Other diagnosed neurodiversity conditions (depression, anxiety, PTSD, bipolar)

Respondents who reported conditions such as depression, anxiety, PTSD, and bipolar disorder showed the lowest overall emotional wellbeing in the AgileBrain assessment, a result driven by a combination of slightly elevated emotional activation and overwhelmingly negative emotional need profiles.

This pattern reflects well-established characteristics of these conditions. Research consistently shows that individuals with mood and trauma-related disorders experience chronic emotional dysregulation, heightened threat sensitivity, and persistent negative affect, all of which contribute to a prevention-oriented motivational style—focusing on avoiding harm or distress rather than pursuing positive outcomes (Beck and Alford, 2009; American Psychiatric Association, 2013).

Furthermore, these respondents demonstrated elevated unmet needs in the self and social domains, such as safety, social support, and fairness, which aligns with findings in trauma literature emphasizing the long-term psychological effects of disrupted attachment and social alienation (Herman, 1992; Etkin and Wager, 2007). These persistent emotional threats and unfulfilled psychological needs understandably result in the lowest wellbeing index across all diagnostic groups studied.

Of the 24 emotional need measures, 6 showed statistically significant differences at the *p* < 0.05 level. In terms of valence, needs are heavily weighted toward prevention (83% negative), suggesting compromised emotional wellbeing. These respondents show a level of activation that is slightly elevated compared to those of OCD and ADHD respondents.

Specifically, individuals reporting other conditions demonstrated significantly higher activation of the following emotional needs:

- **Promotion needs:** Potential (*p* = 0.047)
- **Prevention needs:** Insecurity (negative Safety; *p* = 0.003), Limitation (negative Potential; *p* = 0.004), Stagnation (negative Immersion, *p* = 0.021), Uncared for (negative Caring; *p* < 0.001), Injustice (negative Justice; *p* < 0.001).

Emotional needs are highly concentrated in the domain of the self (50%) and are consistently negative with one exception (Potential). Needs are spread evenly across all three levels of striving (33% each). The largest concentrations of needs occur in *prevention* needs in the domain of the self, and the foundational and experiential levels of striving.

The prevalence of diagnosed psychiatric conditions in this group accords with these findings of poor emotional wellbeing.

## Discussion

### Neurotypical (NT)

#### Emotional activation and wellbeing

Neurotypical (NT) individuals display the highest levels of emotional wellbeing, as evidenced by their significantly lower emotional activation and higher emotional positivity. These results suggest that NT individuals experience fewer emotional challenges compared to those with neurodivergent conditions, as expected. Their emotional needs are better met, leading to a greater sense of satisfaction and stability in their emotional state.

The low emotional activation and high positivity indicate that NT individuals have relatively stable emotional experiences with fewer intense unmet emotional needs. This likely reflects their ability to regulate emotions effectively and their generally favorable social and personal environments, allowing them to manage challenges without becoming overwhelmed. The high wellbeing index supports the idea that NT individuals have a greater capacity to achieve and maintain emotional wellbeing than neurodivergent individuals.

#### Key differences between NT and ND subgroups

The largest gaps between NTs and NDs were found in the unmet needs for Scorn and Exclusion. NTs report substantially lower levels of feeling rejected, judged, or socially marginalized. This indicates that NTs generally experience more stable and secure social relationships, with fewer perceptions of social exclusion or negative evaluation by others. In contrast, NDs report high levels of these unmet social needs, pointing to a significant area of emotional distress within those groups.

In summary, NT individuals report high emotional wellbeing and low levels of unmet emotional needs overall. However, the most striking differences between NTs and NDs appear in Scorn, Exclusion, and Insecurity, with NDs reporting much higher emotional challenges in these areas. This pattern emphasizes that neurodivergent individuals may be particularly vulnerable to feelings of social rejection and emotional insecurity. Although NTs generally have fewer unmet social needs and emotional stability, efforts to support ongoing emotional resilience and meaningful social engagement may help sustain wellbeing.

### Developmental coordination disorder (DCD, dyspraxia)

#### Emotional activation and wellbeing

Individuals with Developmental Coordination Disorder (DCD), also known as Dyspraxia, show a relatively high level of emotional wellbeing, similar to neurotypicals, despite exhibiting higher levels of emotional activation. The relatively low emotional activation observed in DCD participants suggests that their emotional needs, while present, do not create the intense distress seen in other neurodiverse groups, such as those with ADHD or OCD. The DCD group also demonstrated a higher level of positivity, which contributed to their overall high wellbeing index. This suggests that, while DCD individuals may experience emotional activation, they are not negatively overwhelmed by it to the extent seen in other conditions.

Developmental coordination disorder respondents did not demonstrate significant unmet foundational or prevention-oriented needs, which is a notable contrast to the heightened emotional activation in groups like ADHD, where emotional dysregulation is often a primary concern. Rather, the findings indicate that individuals with DCD have a more stable emotional profile, with positive and negative emotional needs distributed across different areas of life. The emotional activation in this group, which is spread across all four domains (self, material, social, and spiritual), implies that while they may experience some emotional challenges, they are not as intensely distressed by them as individuals with conditions like ADHD or OCD.

#### Unmet emotional needs

Developmental coordination disorder participants reported unmet emotional needs primarily in the areas of Authenticity, Conformity, Autonomy, Immersion, Success, Caring, Scorn, Injustice, Ethics, and Purpose. Many of these needs were related to positive, promotional aspects of personal growth, social integration, and self-expression, suggesting that DCD individuals desire more fulfillment in these domains. The unmet need for Authenticity and its symmetrical need for relief from Conformity reflect the internal struggle that individuals with DCD may experience between their authentic selves and societal pressures to conform. This tension could lead to challenges in social integration and self-acceptance, as DCD individuals may feel different from others due to their motor difficulties, which can impact their sense of belonging or self-worth.

**Autonomy and Immersion:** The need for Autonomy and Immersion further underscores a desire for independence and meaningful engagement in activities, despite the challenges posed by their motor coordination difficulties. DCD individuals may seek more control over their actions and environments but struggle to fully immerse themselves in activities due to motor impairments.

**Success:** The unmet need for Success highlights a desire for meaningful achievement in their endeavors, which may be thwarted by the physical limitations that accompany DCD. This can lead to frustration, as success in motor tasks may be more difficult to attain, potentially impacting self-esteem.

**Caring and scorn:** The presence of unmet needs in the Caring and Scorn areas suggests that DCD individuals may desire emotional support and validation but may struggle to receive it in social contexts. This could be due to the challenges DCD individuals face in engaging with others, which can affect how they are perceived socially.

**Purpose:** The unmet need for Purpose indicates that individuals with DCD may struggle with finding a clear sense of direction or meaning, which could be compounded by difficulties in achieving goals and navigating social expectations.

In summary, while individuals with DCD show relatively high emotional wellbeing, they still face challenges related to self-concept, social integration, and personal achievement. Tailored interventions focusing on enhancing their sense of authenticity, autonomy, and success in social and personal pursuits could help address these unmet emotional needs and further improve their emotional wellbeing. Targeted support that emphasizes skill-building, fosters social belonging, and encourages recognition of achievements may be especially beneficial in strengthening resilience within this group.

### Autism spectrum disorder

#### Emotional activation and wellbeing

Individuals with Autism Spectrum Disorder (ASD) exhibit very high levels of emotional activation—both positive and negative—indicating that they experience emotions intensely, even if they may struggle to express or process them in typical ways. This challenges the common stereotype of individuals with ASD being emotionally detached or less affected by emotions. Instead, the findings suggest that ASD individuals feel emotions deeply but may have difficulty navigating and expressing them due to comorbid conditions like alexithymia (Poquérusse et al., 2018), leading to a mix of frustration and emotional dysregulation.

The high emotional activation seen in ASD respondents is coupled with a relatively low wellbeing index, which suggests that the intensity of their emotional experiences can lead to significant distress. This emotional activation appears to be related to a range of needs across different domains, particularly in social and self-related areas, suggesting that challenges in these areas are primary contributors to emotional distress. Despite this, there are also areas of emotional positivity, indicating that individuals with ASD have the capacity for positive emotional experiences, especially when their needs in areas such as autonomy, success, and inclusion are met.

#### Unmet emotional needs

The unmet emotional needs of individuals with ASD span a wide range of domains, particularly in areas related to Authenticity, Conformity, Autonomy, Immersion, Success, Inclusion, Exclusion, Caring, Recognition, Scorn, and Ethics. These needs reflect the emotional struggles that are common among individuals with ASD, such as challenges with social integration, a strong desire for authenticity, and the need for personal autonomy.

**Authenticity and conformity:** The unmet need for Authenticity combined with the related need for relief from Conformity highlights the tension many individuals with ASD feel between being their authentic selves and the pressure to conform to societal norms. ASD individuals often feel different from others due to their social and communication challenges, which can lead to a sense of isolation or frustration when trying to fit into social environments that do not fully accept or accommodate their differences.

**Autonomy and immersion:** The need for Autonomy and Immersion reflects a desire for greater independence and deep engagement in activities, even though ASD individuals may experience difficulties in achieving these desires due to challenges with sensory processing or social interaction. These needs suggest that individuals with ASD have a strong drive for personal agency and meaningful involvement in the world around them, but they may be constrained by their neurological differences.

**Inclusion, exclusion, caring, recognition and scorn:** The social needs of Inclusion, Exclusion, Caring, and Recognition highlight the struggles ASD individuals face in their social relationships. On one hand, they may seek inclusion, care, and recognition; on the other, they may feel excluded or disconnected from others. This tension between wanting to connect and the challenges in doing so can create significant emotional distress. The unmet need for relief from Scorn suggests that ASD individuals may experience feelings of rejection or ridicule, further exacerbating their social struggles.

**Ethics:** The emotional need for Ethics suggests that ASD individuals may have a strong sense of fairness or moral values, which may not always be acknowledged or validated by others. This need for ethical alignment can lead to frustration or distress when individuals feel that the world around them is not in accordance with their values, particularly when they perceive unfairness or injustice in social interactions.

**Recognition:** The unmet need for Recognition reflects the desire for acknowledgment and appreciation, which is often overlooked in individuals with ASD due to difficulties in social communication. This can lead to feelings of being misunderstood or undervalued.

In summary, the emotional wellbeing of individuals with ASD is significantly influenced by their intense emotional experiences, particularly in the areas of social integration, authenticity, and autonomy. While they experience emotions deeply, their challenges in expressing and processing these emotions, coupled with unmet social and personal needs, can lead to emotional distress. Interventions that address these unmet needs—such as promoting social acceptance, enhancing self-expression, fostering autonomy, and reducing social exclusion—could greatly improve the emotional wellbeing and quality of life of individuals with ASD. Focusing support on building authentic social connections and creating environments that respect and nurture individuality may help reduce emotional distress in this population.

### Sensory integration disorder

#### Emotional activation and wellbeing

Individuals with Sensory Integration Disorder (SID) exhibit relatively low levels of emotional activation, indicating that their emotional needs may not be as intensely felt as those in other neurodiverse groups. However, this group’s need profile tends to skew slightly negative, suggesting that although their emotional activation is low, there are still unmet needs that affect their wellbeing. SID participants reported significantly lower fulfillment in the needs for Potential and Ethics, pointing to potential frustration or unmet aspirations regarding personal growth and moral values.

Despite the low emotional activation, the results suggest that SID individuals may still experience challenges in areas related to self-concept and ethical concerns. The lack of significant unmet foundational or prevention-oriented needs further suggests that these individuals do not experience the same level of emotional distress seen in other neurodiverse populations, such as those with ADHD or OCD, where emotional activation is much higher. However, the presence of unmet developmental needs reflects a more subtle, perhaps chronic frustration with unmet personal or ethical aspirations.

#### Unmet emotional needs

**Potential and ethics**: The findings indicate that individuals with SID experience unmet emotional needs primarily in the areas of Potential and Ethics. The unmet need for Potential suggests that individuals with SID may feel that they are not reaching their full capabilities, which could lead to feelings of underachievement or dissatisfaction in their personal growth. The unmet Ethics need highlights a possible internal struggle with moral or ethical concerns, which may be overlooked in traditional assessments of SID but are important for understanding their emotional experience. These unmet needs are less about immediate survival or safety (which may be more prominent in conditions like ADHD or OCD) and more about long-term fulfillment and a sense of integrity.

Importantly, the low levels of emotional activation seen in this group suggest that while the emotional challenges experienced by SID individuals may not be as immediately distressing as those in other conditions, they still reflect important areas of life that could be enhanced with targeted interventions. Fostering environments where SID individuals can explore their full potential and align their actions with their ethical values may alleviate some of these emotional gaps and improve their overall wellbeing.

In summary, while SID individuals may not experience the same level of acute emotional distress as other neurodiverse groups, their unmet emotional needs related to personal potential and ethical concerns point to areas that could benefit from support. Tailored interventions that focus on personal development and ethical alignment could enhance the emotional wellbeing of individuals with SID. Encouraging sensory-friendly environments that nurture growth and align with personal values may be particularly effective in supporting long-term wellbeing for this group.

### Sensory hypersensitivity (SH)

#### Emotional activation and wellbeing

Participants with Sensory Hypersensitivity (SH) exhibit a high level of emotional activation, similar to individuals with ASD, ADHD and OCD, but with a notable skew toward negative (prevention-oriented) emotional needs. This heightened emotional activation suggests that individuals with SH experience greater emotional intensity, which may be linked to the overwhelming sensory experiences they face. However, despite the high activation, SH participants still demonstrate a somewhat positive emotional need profile, which is not as intensely negative as those in conditions like OCD or ADHD. This suggests that while SH individuals may experience significant emotional stress due to sensory overload or hyperawareness, they still retain an ability to experience some degree of emotional positivity.

The emotional activation in SH individuals is spread across multiple domains, including the self, material, social, and spiritual areas, indicating that their emotional challenges are multifaceted. While they may experience sensory-related stress, their emotional needs also span other aspects of life, reflecting the complexity of their emotional experience. The balance of positive and negative emotional needs in SH participants suggests that while they may struggle with sensory input, they also have the capacity for positive emotional experiences when their needs are met in certain areas.

#### Unmet emotional needs

The unmet emotional needs for SH individuals are spread across various domains and levels of striving. The most significant unmet needs are related to Insecurity, Authenticity, Autonomy, Success, Inclusion, Exclusion, Caring, Scorn, Ethics, and Purpose. These needs suggest that individuals with SH experience a significant amount of emotional vulnerability, particularly related to their sense of security, social belonging, and self-expression.

**Insecurity, authenticity, and conformity:** The unmet need for Insecurity indicates that individuals with SH often feel a heightened sense of vulnerability, especially in social and sensory environments. This aligns with the experience of sensory overload, which can contribute to a feeling of being overwhelmed or unsafe in various settings. Similarly, the unmet need for Authenticity and the associated for relief from Conformity pressures suggest that SH individuals may feel pressure to conform to social expectations, which conflicts with their authentic self, further contributing to emotional distress.

**Autonomy and success:** These needs reflect a desire for independence and achievement. While SH individuals may wish to engage more fully in activities, the sensory overload they experience can make it difficult to gain mastery over their environment or achieve success in tasks, leading to frustration and dissatisfaction.

**Inclusion and exclusion:** The unmet social needs, particularly around Inclusion and Exclusion, highlight the challenges SH individuals face in social interactions. They may struggle to feel accepted or understood in social contexts, contributing to feelings of isolation or rejection.

**Scorn:** The negative emotional experience of Scorn points to a desire for emotional support that is often unmet, leading to a sense of neglect or being undervalued in social environments.

**Ethics and purpose**: Additionally, the unmet need for Ethics suggests that SH individuals may have strong internal moral values but feel conflicted when these values are not reflected in their experiences. This internal tension can further contribute to emotional distress. The unmet need for Purpose indicates a potential struggle in finding meaning or direction, which may be exacerbated by the sensory challenges they face in their everyday lives.

In summary, individuals with Sensory Hypersensitivity experience significant emotional activation due to their heightened sensitivity to sensory stimuli, coupled with unmet emotional needs across various domains. These needs reflect challenges in achieving emotional security, social integration, and personal success. Interventions that focus on reducing sensory overload and enhancing social inclusion, while also fostering personal autonomy and achievement, could greatly improve the emotional wellbeing of individuals with SH. Supporting this population with sensory-friendly environments and targeted social supports may help mitigate emotional distress and improve daily functioning.

### Obsessive compulsive disorder

#### Emotional activation and wellbeing

Individuals with Obsessive-Compulsive Disorder (OCD) display moderate levels of emotional activation, similar to those seen in ADHD, but their emotional needs are predominantly negative (prevention-oriented). This suggests that OCD participants experience emotional distress not only due to the intensity of their feelings but also due to their focus on avoiding negative outcomes and preventing perceived threats. Their emotional need profile leans heavily toward the negative side, indicating that OCD individuals tend to be more affected by emotional distress than by positive emotional experiences.

The overall wellbeing index for OCD respondents is relatively low, reflecting the high levels of emotional activation combined with their negative emotional needs. This is consistent with the nature of OCD, where intrusive thoughts and compulsive behaviors lead to constant emotional turmoil and a persistent need to prevent harm or discomfort. Emotional activation in OCD participants is primarily focused in the social and material domains, suggesting that their emotional distress is largely related to their social interactions and the perceived need for control over their external environment.

#### Unmet emotional needs

The unmet emotional needs of individuals with OCD span a variety of domains, with significant gaps in Insecurity, Limitation, Autonomy, Immersion, Success, Exclusion, Uncared for, Scorn, and Injustice. These needs align with the emotional struggles commonly faced by individuals with OCD, such as difficulties with control, autonomy, and self-worth.

**Insecurity, limitation, and autonomy:** The unmet need for Insecurity, reflects the core anxiety and fear that underpins OCD. OCD individuals often engage in compulsive behaviors to alleviate the persistent fear of harm or contamination, leading to a heightened sense of insecurity. This is compounded by unmet needs for Limitation and Autonomy, suggesting that OCD individuals feel constrained in their actions, unable to exercise control over their environment or behavior, which fuels their compulsions.

**Immersion and success:** The need for Immersion and Success indicates that individuals with OCD may long for greater engagement and achievement in their daily lives but are hindered by the intrusive thoughts and compulsions that limit their ability to fully immerse themselves in tasks or experience success in a meaningful way. Their compulsive behaviors often prevent them from completing tasks in a satisfying or productive manner, leading to frustration and a sense of failure.

**Exclusion, uncared for and scorn:** The unmet social needs, such as Exclusion, Uncared for, and Scorn, point to the isolation and misunderstanding that OCD individuals may experience in their relationships. Their compulsions and rigid behaviors can strain social connections, leading to feelings of rejection or neglect. This aligns with the challenges of social integration often observed in OCD, where the compulsions create barriers to authentic social engagement and emotional connection.

In summary, the emotional wellbeing of individuals with OCD is significantly impacted by their need for safety and control. Their emotional needs are largely negative and reflect a pervasive sense of insecurity, frustration, and social disconnection. Addressing these unmet emotional needs through therapeutic interventions that focus on reducing compulsions, fostering social connection, and promoting a sense of autonomy and control could help alleviate the emotional distress experienced by individuals with OCD. Interventions that strengthen coping strategies, enhance emotional regulation, and promote meaningful social connections may offer the greatest benefit to this population.

### Attention deficit hyperactivity disorder (ADHD)

#### Emotional activation and wellbeing

Individuals with Attention Deficit Hyperactivity Disorder (ADHD) display moderate emotional activation, similar to those with OCD, but with a more pronounced negativity in their emotional needs. This suggests that while ADHD individuals experience heightened emotional intensity, particularly in response to frustration or overstimulation, their emotional experiences tend to skew negative. The emotional distress they face is compounded by their struggles with emotional regulation, which is a hallmark of ADHD.

The overall wellbeing index for ADHD respondents is notably lower than that of neurotypicals, but not as low as individuals with psychiatric disorders such as depression, bipolar disorder, or PTSD. This reflects the balance of both positive and negative emotional needs—ADHD participants express desires for Success, Autonomy, and Immersion, but these desires are often unmet due to impulsivity, distractibility, and challenges with sustained focus. The relatively high levels of activation in the social and self domains indicate that ADHD individuals feel the intensity of their emotional needs particularly in personal and interpersonal settings, where their impulsivity or struggles with attention may interfere with achieving positive emotional outcomes.

### Unmet emotional needs

The unmet emotional needs of individuals with ADHD are primarily concentrated in the areas of Insecurity, Conformity, Autonomy, Immersion, Success, Exclusion, Uncared for, Scorn, Justice, Ethics, and Purpose. These needs highlight the emotional challenges ADHD individuals face, particularly in terms of self-esteem, achieving personal goals, social relationships, and living in accordance with higher principles.

**Insecurity:** The unmet need for relief from feelings of Insecurity suggests that ADHD individuals may feel emotionally vulnerable due to their struggles with impulsivity and lack of focus. This can create a sense of instability, where they may feel unsafe in their environment or social relationships due to their difficulties with attention, self-regulation, and maintaining focus on tasks or interactions.

**Conformity:** The need for relief from Conformity reflects the internal struggle ADHD individuals face between adhering to societal expectations and remaining true to their authentic selves. Many ADHD individuals find it challenging to meet social or academic norms, leading to frustration and the feeling that they cannot express themselves fully in a world that may not accommodate their neurodivergence.

**Autonomy and immersion:** The unmet need for Autonomy and Immersion speaks to a desire for greater independence and deeper engagement in activities. ADHD individuals often feel a lack of control over their behavior and environment, particularly in structured settings like work or school, where attention and focus are critical. These unmet needs contribute to feelings of powerlessness or frustration, as ADHD individuals may struggle to stay engaged in tasks or complete goals without external structure and support.

**Exclusion, uncared for, and scorn:** The need for relief from social Exclusion, feeling Uncared for, and Scorned indicate that ADHD individuals may feel rejected, neglected, or misunderstood in social interactions. Their impulsivity or distractibility may lead to miscommunications or social difficulties, causing them to feel excluded or unappreciated by others. These social struggles, combined with difficulties in maintaining friendships or connections, contribute to emotional distress and isolation.

**Justice and ethics:** The unmet need for Justice and Ethics highlights ADHD individuals’ strong sense of fairness and moral integrity, which is often not fulfilled in environments that value organization and structure over spontaneity and creativity. This misalignment between their needs and the expectations placed upon them can lead to frustration and a sense of injustice, especially when they feel that their actions are misunderstood or unfairly judged by others.

**Purpose:** The unmet need for Purpose reflects a desire for meaningful engagement and direction in life. ADHD individuals may struggle to find a sense of purpose or direction due to their difficulties with sustained attention and long-term planning, often feeling lost or unsure of how to reach their goals.

In summary, the emotional wellbeing of individuals with ADHD is significantly affected by their challenges with emotional regulation, attention, and social integration. Their unmet emotional needs span all four life domains, reflecting their struggles with insecurity, autonomy, and social exclusion. Interventions that focus on improving emotional regulation, enhancing self-esteem, and providing structures for social and academic success could help address these unmet needs and improve the emotional wellbeing of individuals with ADHD. Targeted strategies that promote autonomy, personal achievement, and supportive social environments are especially critical for fostering resilience in this group.

### Other diagnosed neurodiversity conditions (depression, anxiety, PTSD, bipolar)

#### Emotional activation and wellbeing

Individuals in this category—those diagnosed with depression, anxiety, PTSD, bipolar disorder, or other psychiatric conditions—report the lowest overall emotional wellbeing of all groups studied. Their emotional activation is predominantly negative and prevention-oriented, reflecting a strong focus on avoiding harm, distress, or further emotional pain. This high level of negative emotional activation aligns with the well-established emotional challenges faced by these populations, where experiences of insecurity, fear, hopelessness, and emotional dysregulation are common.

The emotional activation pattern suggests that individuals in this group are heavily influenced by their internal emotional states, which often center on feelings of vulnerability and threat. Unlike other groups, positive, promotional strivings are largely crowded out by relief needs, limiting motivational energies that might be directed toward the pursuit of joy or fulfillment. This imbalance significantly contributes to their low wellbeing index and highlights the chronic emotional strain these individuals endure.

#### Unmet emotional needs

The unmet emotional needs of this group reflect a profound struggle with safety, personal growth, and social connection. Specifically, significant unmet needs include Insecurity, Potential, Limitation, Stagnation, Uncared for, and Injustice. These needs span all four life domains, underscoring the complexity of their emotional challenges.

**Insecurity:** The strong unmet need for relief from feelings of Insecurity illustrates the deep emotional vulnerability experienced by these individuals. Many may live with a persistent sense of threat, fear, or emotional instability, whether due to anxiety, trauma, or mood fluctuations. This need for safety is foundational and its absence contributes heavily to ongoing emotional distress.

**Potential, limitation, and stagnation:** Unmet aspirational needs such as Potential and relief from feelings of Limitation reveal feelings of being unable to grow or develop. Individuals in this group may struggle with self-doubt, hopelessness, or the belief that their circumstances prevent them from realizing their capabilities. This is further compounded by significant Stagnation, indicating a lack of progress or forward momentum in life—an emotional experience often described by individuals with depression or trauma-related conditions.

**Uncared for:** The social domain is also notably impacted, with significant unmet needs around Uncared for, pointing to feelings of isolation, abandonment, or a lack of emotional support. Individuals in this group may perceive that others are indifferent to their struggles, intensifying feelings of loneliness and disconnection.

**Injustice:** The need for relief from feelings of Injustice reflects a perception of unfairness or moral injury—whether from life experiences, trauma, or societal treatment—which can deeply affect emotional wellbeing. Feelings of being wronged or that life is unfair are common in these conditions and can perpetuate cycles of anger, resentment, or despair.

In summary, individuals reporting other psychiatric conditions such as depression, anxiety, PTSD, and bipolar disorder exhibit the most negative emotional need profiles and the lowest wellbeing index in this study. Their emotional landscape is dominated by prevention-oriented needs focused on avoiding harm, emotional pain, and continued personal stagnation. This group’s profile reflects profound emotional vulnerability, chronic distress, and significant social isolation.

Therapeutic interventions that prioritize emotional safety, reduce social isolation, and foster long-term personal growth may be essential for improving wellbeing in this group. Particularly, interventions that focus on rebuilding trust, supporting social reintegration, and fostering hope and personal development may offer critical support for these individuals. Empowering this population to reconnect with their potential and build secure, caring relationships could help mitigate emotional distress and promote healing.

#### Overall summary of emotional needs across groups

The patterns of unmet emotional needs across neurodivergent conditions discussed above and summarized in Tables 6–8, reveal important distinctions. A common feature is the concentration of prevention needs in the self and social domains, suggesting that issues related to self-concept and social relations are widespread across neurodivergent conditions. In contrast, needs in the material domain tend to focus on promotion or growth, highlighting how individuals with neurodivergent conditions often direct their striving toward achievement and independence as coping mechanisms. These individuals face challenges in building social relationships, which can lower self-concept and make material needs more prominent, as they can be met with less reliance on social competence or self-esteem.

Although all of these conditions are classified under the umbrella of neurodiversity, it’s crucial to recognize the differences in how each manifests. There are significant variations in the emotional toll, stress (activation), outlook, and unmet needs across conditions. For example, ADHD and OCD may involve higher emotional activation and distress, while ASD is associated with greater emotional challenges related to social inclusion and authenticity. These differences emphasize the need to acknowledge each condition’s unique emotional needs profile (Tables 6–8).

Recognizing these distinctions is essential for developing individualized interventions. A one-size-fits-all approach will not address the specific challenges each group faces. Tailored strategies that meet both deficiency and growth needs will be key to supporting emotional wellbeing for neurodivergent individuals.

Although we have analyzed and reported on differences by condition in aggregate, it’s important to recognize that each individual has a unique set of aspirations and challenges. Therefore, there is no substitute for individualized assessment using tools like AgileBrain. In general, we recommend integrating an AB assessment into wellness, wellbeing, and health systems and providing users with their AB results alongside relevant available resources, to ensure immediate therapeutic intervention and mental health support for those showing signs of distress.

## Implications

Findings from this study provide evidence for AgileBrain’s construct validity as a tool for assessing emotional wellbeing among neurodivergent workers. In these results, AgileBrain scores effectively differentiated wellbeing levels among participants with and without diagnosed neurodivergent conditions, demonstrating its potential for identifying emotional wellbeing challenges within this population. Additionally, given the preference for brief, engaging, and gamified digital assessments (Newson et al., 2020; Pincus, 2024d), AgileBrain offers a scalable and accessible approach to assessing wellbeing in neurodiverse workplace populations.

## Strengths and limitations

A key strength of this study is its use of a large, population-representative sample of full-time employees, allowing for an examination of emotional wellbeing differences among neurodivergent and neurotypical workers. This sampling strategy highlights AgileBrain’s potential as a practical and scalable screening tool for organizations. However, a primary limitation is that the study is cross-sectional, providing only a snapshot from a single wave of data collection in June 2023 rather than tracking changes over time. Future longitudinal research is needed to evaluate AgileBrain’s ability to detect wellbeing fluctuations within neurodivergent employees and workplace settings.

## Conclusion

This study offers initial evidence supporting AgileBrain’s validity in assessing emotional wellbeing within neurodiverse workplace populations. AgileBrain demonstrated sensitivity to wellbeing differences across employees with various neurodivergent conditions, suggesting its utility as a non-intrusive screening tool for workplace wellbeing assessments. Given the growing emphasis on neurodiversity inclusion and the preference for gamified, digital assessments, AgileBrain provides a viable alternative to traditional self-reports for organizations seeking to support their neurodiverse workforce.

Importantly, beyond its ability to measure wellbeing, this approach can tell us “what to do about it.” AgileBrain is able to identify specific unmet needs that can be addressed in order to support and build resilience in workers with compromised emotional wellbeing. This diagnostic and prescriptive capability is unique among wellbeing assessments to our knowledge.

These findings underscore the critical importance of advancing research into the emotional wellbeing of neurodivergent individuals—particularly within industries like software development, IT, engineering, biotechnology, and academia, where neurodiversity is both prevalent and increasingly recognized as a strength. Estimates suggest that as many as 34% of people in STEM fields identify as neurodivergent (Wei et al., 2015), and in biotechnology specifically, the number reaches nearly one in four (24%) according to the 2023 *Employee Engagement and Retention in Biotechnology* report by Keystone Partners and Leading Indicator Systems (Keystone Partners, 2025). This substantial segment not only shows distinct emotional needs—such as heightened desires for caring, safety, autonomy, and justice—but also tends to rate their employers more favorably when those needs are met. Neurodivergent employees are significantly more likely to feel greater appreciation for ethics-driven leadership. Notably, they respond positively to specific management practices like extra support during role transitions (+30 points), targeted coaching (+16), and regular personal check-ins (+19).

As formal diagnoses of neurodivergent conditions such as ADHD, autism, OCD, and others continue to rise, organizations must recognize that neurodiversity is not limited to younger generations. While Gen Z is driving much of the current conversation, many adults—particularly in Gen X and older—are now receiving diagnoses later in life, often after their children are evaluated and diagnosed. This growing recognition reflects both greater awareness of hereditary patterns and the historic underdiagnosis of these conditions, especially in high-performing individuals within fields like software development, engineering, biotechnology, and academia.

These insights not only deepen our understanding of emotional wellbeing in neurodiverse populations but also have powerful implications for workplace integration. AgileBrain’s ability to identify specific unmet emotional needs—such as the desire for autonomy, recognition, or psychological safety—equips organizations with actionable data to reshape training, management, and coaching practices. By tailoring workplace environments and support systems around these insights, employers can create more inclusive and emotionally supportive cultures that extend across all career stages.

This is not only an ethical imperative but a strategic one—essential for retaining top talent, supporting late-diagnosed professionals, and maximizing engagement in sectors where neurodiversity often drives innovation. AgileBrain can inform more inclusive onboarding, targeted leadership development, and individualized coaching, enabling organizations to move beyond inclusion toward full integration—where neurodivergent individuals are empowered to thrive, feel understood, and contribute meaningfully. In industries like technology, biotech, and academia, where neurodivergent individuals are frequently overrepresented, such approaches are critical to both human wellbeing and organizational success.

## Data Availability

The raw data supporting the conclusions of this article will be made available by the authors, without undue reservation.

## Footnotes

^1^Wellbeing is not hyphenated when referring to the AgileBrain Wellbeing Index.
